# New artificial hematophagy system with attractive polymeric biofilm for maintenance of *Culex quinquefasciatus* (Diptera: Culicidae) in the laboratory

**DOI:** 10.1186/s13071-024-06162-3

**Published:** 2024-03-15

**Authors:** Angelita Milech, Caroline Quintana Braga, Carolina dos Santos Bermann, Jaqueline Ferreira de Souza, André Ricardo Fajardo, Élvia Silveira Vianna, Camila Belmonte Oliveira

**Affiliations:** 1 Department of Microbiology and Parasitology, Institute of Biology, Federal University of Pelotas (UFPel), Pelotas, RS Brazil; 2 Center for Sciences Chemical, Pharmaceutical and Food Sciences, Federal University of Pelotas (UFPel), Pelotas, RS Brazil

**Keywords:** Mosquitoes, Blood feeding, Attractive membrane, *Culex quinquefasciatus*

## Abstract

**Background:**

Maintaining mosquito colonies in the laboratory requires a blood supply so that females' oocytes can mature and oviposition can take place. In this study, a new artificial hematophagy system for colonization and maintenance of *Culex quinquefasciatus* in the laboratory was developed and tested.

**Methods:**

We developed an attractive polymeric biofilm including 25% l-lactic acid for use as a membrane in an artificial hematophagy system and compared the feeding rate of females with Parafilm-M^®^. We also evaluated the oviposition rate, larval survival and adult emergence of females fed through the attractive biofilm.

**Results:**

The average percentage of female *Cx. quinquefasciatus* fed through the attractive biofilm was 87%, while only 20% became engorged with Parafilm-M^®^ (*p* < 0.0001). Feeding through the attractive biofilm developed in this study produced high levels of evaluated biological parameters; the percentage of egg laying by females that underwent artificial hematophagy through the biofilm was 90%, with an average of 158 eggs per raft. From these eggs, 97% of the larvae hatched, of which 95% reached the pupal stage. The adult emergence rate corresponded to 93% of pupae.

**Conclusions:**

Insects fed with attractant through the biofilm system had a higher engorgement rate compared to those fed through Parafilm-M^®^. Our study is preliminary and suggests that polymeric biofilm has great potential for artificially feeding mosquitoes in the laboratory. Based on this research, new studies will be carried out with biofilm and different systems.

**Graphical Abstract:**

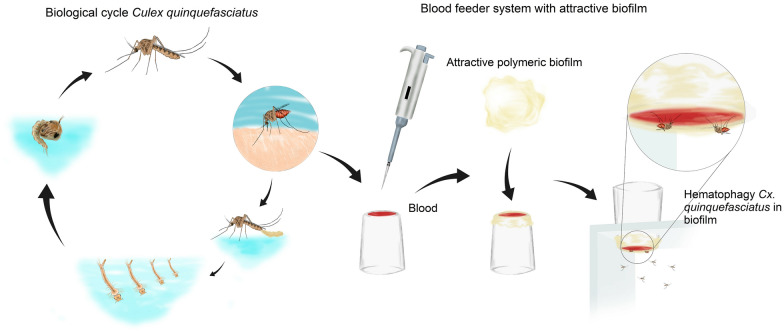

## Background

The mosquito *Culex quinquefasciatus* Say, 1823 (Diptera: Culicidae), is an important vector of pathogens such as the *Wulchereria bancrofti* and West Nile virus [1]. It is also a potential vector of the arbovirus responsible for Saint Louis encephalitis [2]. Recently, in Brazil, specimens of this insect from urban environments were found to be infected with Zika virus (ZIKV), suggesting participation in a new cycle of this arbovirus [3, 4]. This mosquito species has a wide geographic distribution in urban and suburban areas from tropical to temperate zones. In addition, it exhibits opportunistic blood-feeding behavior in different hosts, and its ability to transmit zoonotic disease agents poses a risk for possible cross-breeding between wild bird arboviruses and spread to humans, especially in urban areas [4–6].

Culicidae females, in general, need to perform hematophagy for the maturation of their oocytes and subsequent egg development [6]. The blood supply of vertebrates is an essential step and represents the greatest challenge for the colonization and maintenance of these insects in the laboratory. Laboratory animals are often used as a source of blood; however, there are a number of restrictions and ethical limitations to this method, in addition to the high cost and need for a specialized professional to maintain the animals in the vivarium [7]. The maintenance of mosquito species in the laboratory, as well as other arthropods of public health importance, is essential for studies on biology, behavior and the development of control techniques.

Artificial feeders have been developed and routinely used in procedures to maintain mosquitoes in a laboratory environment [8]. These feeders are usually composed of one or more blood reservoirs, usually covered by thin membranes of animal tissue, Parafilm-M^®^ or collagen [9], which facilitate the penetration of female mouthparts for food. To simulate the presence of a host and attract mosquitoes, heating methods are used [8]. Hemotek membrane feeders (Discovery Workshops, Accrington, UK) use an electrical heating element to maintain the temperature of the blood meal [10]. The glass feeder has an external area with warm water circulation and an internal chamber where the blood is spilled [11]. However, some of these methods are expensive, time-consuming and subject to regulation and inspection, thus limiting their use in many laboratory settings [12]. Therefore, the development of feeding systems capable of overcoming these disadvantages is necessary.

Implementation of biopolymers in artificial feeding systems is little explored, although their use in the food and biomaterials area is widespread. Gelatin (Gel), a biopolymer derived from collagen, is widely used in various industries because of its non-toxicity, biodegradability and biocompatibility properties [13]. To improve the filmogenic properties of these biopolymers, it is common to combine them with synthetic polymers [14]. Poly (vinyl alcohol) (PVA) is a biodegradable, water-soluble and non-toxic synthetic polymer widely used in film production [15]. l-Lactic acid is one of the main volatiles secreted by the eccrine sweat glands of the skin and exhalation. This was the first substance isolated and identified to have an attractive effect on mosquitoes in laboratory behavioral tests [16]. In double-port olfactometer studies, *Cx. quinquefasciatus* was attracted to l-lactic acid [17, 18]. Therefore, this substance can be used in technologies that improve the attractiveness of artificial feeding models used in the laboratory to mosquitoes. Therefore, the objective of this study was to develop an artificial hematophagy system and test for the colonization and maintenance of *Cx. quinquefasciatus* in the laboratory, using a polymeric biofilm of Gel and PVA, adding l-lactic acid to its composition, as an agent for attraction to and induction of blood meals.

## Methods

### Colony establishment and maintenance

To establish the colony of *Cx. quinquefasciatus* in the laboratory, initially a field collection of *Culex* spp. eggs was carried out, arranged in the form of a raft under water, using an entomological apparatus and glass containers. The collection was carried out in the urban area of ​​the city of Pelotas, Rio Grande do Sul, Brazil (31°46ʹ28.4″S/52°21ʹ07.0″W). Subsequently, the samples were sorted, and the egg rafts were placed in trays containing dechlorinated water, kept at a temperature of 25 ℃ for the hatching of the larvae. These were identified by sampling each tray by dichotomous key [6]. After confirming the species, the larvae were fed wet kitten chow (0.5 to 1 *g*/tray) every 2 days, and the water was changed once during the larval stage. After reaching the pupal stage, they were transferred to cages identified with the species, date and generation number (F.0 for specimens collected in the field) for colony control. The cages used were made of plastic material (30 × 20 × 20 cm) with screens on the top and sides for air circulation and a top opening with access to the feeding apparatus. After emergence of adults, 10% sugar solution was added ad libitum.

In all stages, the environment was maintained under controlled conditions of 12-h light/dark photoperiod, relative humidity (RH) 60% to 70%, temperature 25 ℃ ± 2 ℃ and a screened exhaust system.

### Preparation of the polymeric biofilm

The biofilm was prepared by conventional casting technique [19]. Initially, an aqueous solution of gelatin 1.3% (*w*/*v*) was prepared, which was kept under magnetic stirring at room temperature for 1 h. In parallel, an aqueous solution of poly (vinyl alcohol) (PVA) 1.3% (*w*/*v*) was also prepared and kept under magnetic stirring for 1 h at 80 ℃. Then, the two solutions were mixed and homogenized for 30 min at room temperature. Soon after, an amount of glycerol equivalent to 40% of the mass of the polymers was added to the mixture to act as a plasticizer. To make the biofilm attractive to blood-sucking insects, l-lactic acid (25% of the total mass of the system) was added to the filmogenic solution. Finally, the solution resulting from this process was poured into a Petri dish and transferred to an oven at 37 ℃ for 48 h for the complete evaporation of the solvent and biofilm formation. The attractive polymeric biofilm is part of the artificial hematophagy system that we developed and produced, and this was published with the INPI under patent number (BR 102021015280-0 A2).

### Analysis of the mechanical properties of the biofilm

The mechanical properties of the prepared biofilm (rupture strength, maximum elasticity and Young's modulus) were determined from stress tests as described by Voss et al. [20]. The fracture behavior of biopolymer-based materials is of great economic concern as it affects the resistance of these materials during handling, processing and transportation, for example. Young’s modulus determines the stiffness of a material, the relationship between stress and strain. Therefore, an investigation of these properties is important. To determine the influence of EDTA on the polymer matrix, mechanical tests were carried out before and after immersion of the biofilm in the EDTA solution [20]. The tests were carried out with texturometer equipment (Stable Microsystems TA.XT2 texturometer, UK) using rectangular specimens (26 mm long, 76 mm wide and 0.18 mm thick). The analysis parameters followed the ASTM D882-12 standard, and the tests were performed with four replicates (*n* = 4) using samples before and after exposure to the EDTA solution (1% *w*/*v*).

### Artificial hematophagy system

**Fig. 1:**
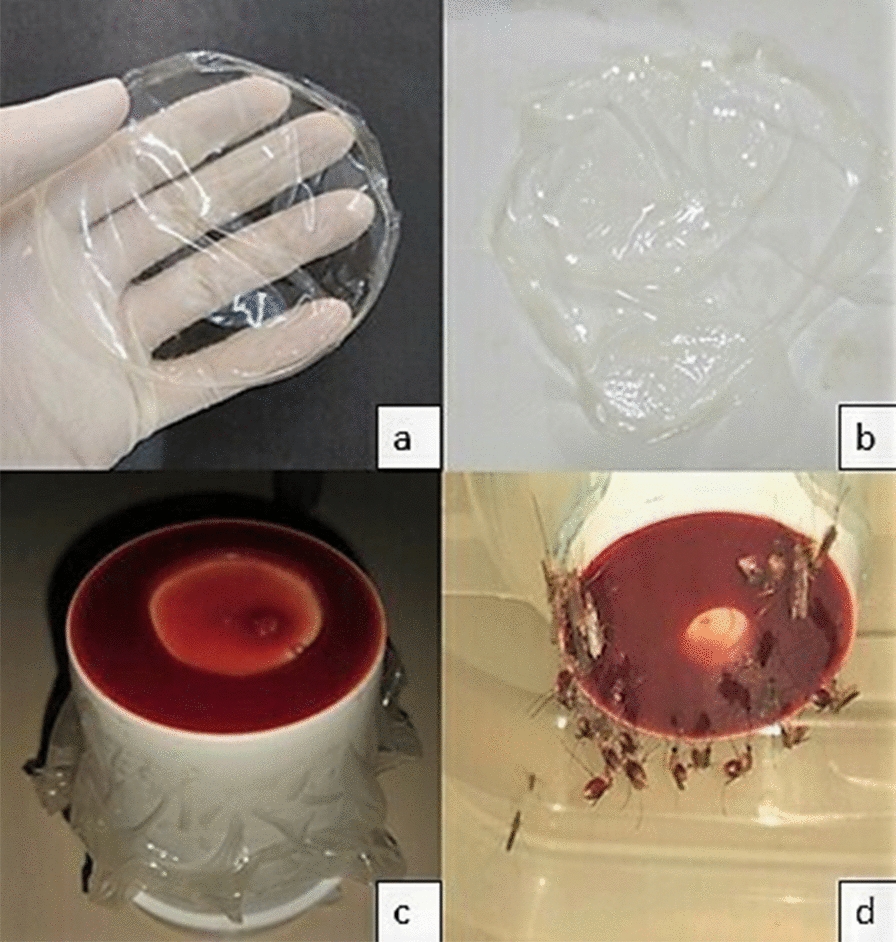
Assembly process and use of the artificial hematophagy system. (**a**) Attractive polymeric biofilm; (**b**) attractive polymeric biofilm after treatment with 1% EDTA (**c**); ready-to-use artificial hematophagy system; (**d**) *Culex quinquefasciatus* females taking the blood meal through the biofilm

The artificial hematophagy system was based on the method described by Rutledge et al. [21], composed of a plastic cup with a small external cavity at its bottom to house the blood and covered with biofilm or Parafilm-M^®^, forming the feeding surface. To use the biofilm (Fig. 1a), it had to be immersed in a 1% ethylenediaminetetraacetic acid (EDTA) solution for 1 min (Fig. 1b). EDTA is a very common anticoagulant for this type of process; since the polymers used are extremely hydrophilic, EDTA acts by creating a physical barrier between the blood and polymeric matrix, preventing the biofilm from absorbing the blood, making the food accessible to the insect [22]. Blood was collected from bovine animals housed at the Veterinary Hospital of the Federal University of Pelotas (HUV/UFPel) by puncturing the jugular vein in a BD Vacutainer^®^ tube with EDTA K2. Approximately 2 ml of the blood sample was transferred to the external cavity of the cup using a pipette. Then, the biofilm was spread over the cavity, staying in contact with the blood, and fixed to the outer side of the cup with double-sided tape (Fig. 1c). Water (37 ℃) was added to the inner cavity of the cup to keep the blood at an ideal temperature for hematophagy (Fig. 1d). For validation of this system, the apparatus was also made with the method using Parafilm-M^®^, which was rubbed on human skin for adhesion of natural odor binders [23].

### Assay

For the artificial hematophagy test, two treatments were performed, sorted into the following groups: (i) artificial hematophagy system with polymeric biofilm with 25% l-lactic acid; (ii) artificial hematophagy system with Parafilm-M^®^. The two feeding trials were conducted simultaneously using three replicates of cages, each containing 20 mosquitoes per cage for each treatment (*n* = 60) [24, 25], with an average age of 5 days. These mosquitoes originated from a cage of males, with a 1:1 male-to-female ratio of *Cx. quinquefasciatus* of the same age. Feeding occurred over a 1-h period in a controlled dark environment with a temperature of 25 ℃. Three days after blood-feeding, a dark container with dechlorinated water was introduced into each cage to facilitate egg-laying by the females. Feeding lasted 1 h in a dark environment with controlled temperature (25 ℃). Three days after hematophagy, a dark container with dechlorinated water was placed inside each cage so that the females could lay eggs. The fecundity rate was assessed through the oviposition of females after 4 to 6 days.

### Evaluation of biological parameters

The engorgement rate was determined by collecting observational data. A fully engorged mosquito was identified by visually inspecting the fully distended and darker colored abdomen [26]. All fed females were considered fully engorged, and none were removed from their respective groups. The oviposition rate was assessed by counting the number of rafts (clusters of eggs) and dividing it by the number of engorged females. To evaluate other biological parameters, five rafts with dozens of eggs from each cage (*n* = 15) were selected and individually placed in plastic trays with dechlorinated water for the eggs to hatch. The average number of eggs produced per female was determined by counting the eggs per raft using a stereomicroscope. After hatching, the larvae were kept in trays and fed daily with macerated wet kitten food. After 48 h, both the larvae and their pupae were quantified. Subsequently, these pupae were transferred to cages to assess the adult emergence rate.

### Statistical analysis

Statistical analysis was performed using GraphPad Prism version 9.0 for Windows, Graph Pad Software (San Diego, CA, USA). One-way analysis of variance (ANOVA) followed by Tukey's test was used to determine whether there were statistically significant differences (*p* < 0.05) between the artificial blood-feeding methods.

## Results

The polymeric biofilm was translucent; when stretched, the thickness varied from 0.10 to 0.12 mm. This thickness represents greater flexibility and stability. After immersion in 1% EDTA solution (*w*/*v*) and being extended again, the average thickness reduced to 0.05 to 0.07 mm, making the biofilm even more flexible. Furthermore, the biofilm with EDTA showed good mechanical properties, where the maximum rupture stress and Young's modulus were slightly higher compared to the sample without EDTA (Table 1). Regarding elasticity, no statistically significant changes were observed for either sample (with or without EDTA). We noticed that the EDTA biofilm also became more hydrophobic, which could be an advantage in terms of handling and practicality of application.

**Table 1:** Mechanical properties determined from the tensile test for samples of biofilm before and after exposure to 1% EDTA solution (*w*/*v*)

Sample	Breaking force (*N*)	Maximum elongation (%)	Young’s modulus (kPa)
Biofilm without EDTA	1.1 ± 0.1	63.42 ± 8.03	3.84 ± 0.16
Biofilm with EDTA	2.2 ± 0.8	70.32 ± 12.34	6.38 ± 0.27

**Fig. 2:**
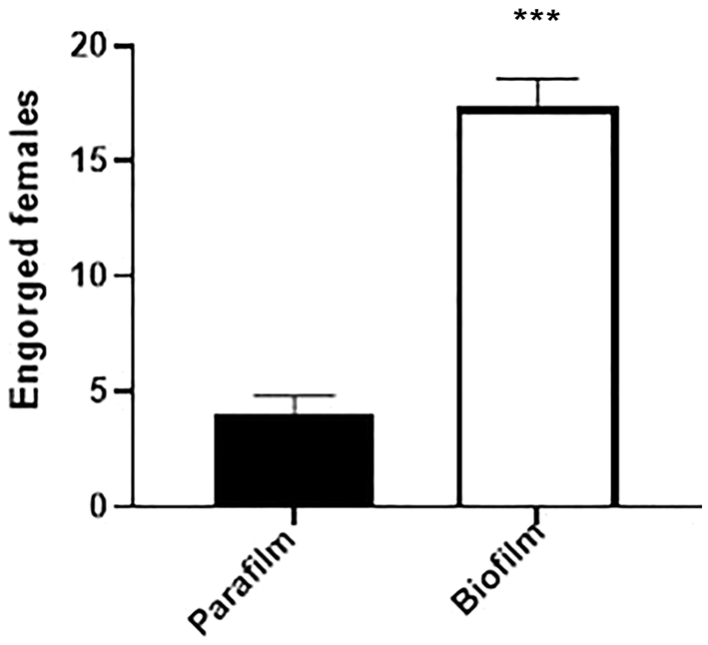
Number of engorged females after artificial hematophagy on biofilm and Parafilm-M^®^. *Statistical difference by the ANOVA test followed by Tukey’s test (*p* < 0.0001)

The average percentage of caged *Cx. quinquefasciatus* mosquitoes (60 mosquitoes/cage) artificially fed using an attractive biofilm system was 87%, while only 20% became engorged with Parafilm-M^®^. There was a statistically significant difference between the different membranes used (*p* < 0.0001), and the data are shown in Fig. 2. The laying rate of females that performed artificial hematophagy through the biofilm was 90% with an average of 158 eggs per female. There was no laying by females fed through Parafilm-M^®^, so their laying rate was 0%. Therefore, we continued to evaluate the viability in relation to the hatching and development of larvae, pupae and adults only from group A. The percentage of larvae that hatched was 97%, of which 95% reached the pupal stage. The adult emergence rate corresponded to 93% of pupae. At the end of the cycle, each female fed through the biofilm was capable of generating 134 adults.

## Discussion

Maintaining mosquito colonies in the laboratory requires a blood supply for females to mature their oocytes and develop eggs [6]. Due to current bioethical standards, the direct use of animals is being replaced by artificial blood-feeding. Large-scale mosquito breeding laboratories commonly employ Hemotek^®^, specific equipment for artificial blood-feeding (Hemotek Ltd., Blackburn, UK) [27]. This method is expensive to implement and requires infrastructure and supervision [28]. The present study is innovative in describing the development and applicability of an attractive membrane for hematophagous insects, utilizing easily acquired components capable of attracting and inducing *Cx. quinquefasciatus* to partake of an artificial blood meal under laboratory conditions at a lower cost. This innovation involves the development of a biofilm using readily available components such as gelatin, polyvinyl alcohol and lactic acid, offering a cost-effective solution for attracting and inducing the artificial blood meal of *Cx. quinquefasciatus* under laboratory conditions.

Different membranes have been used in artificial feeding procedures in an attempt to simulate the skin of vertebrate animals. Some studies have indicated a predilection of mosquitoes for natural membranes, but there are reports that other synthetic membranes, such as intestinal collagen and Parafilm-M^®^, have been successful with some Culicidae species [11, 21, 29]. The polymeric biofilm developed in this study is composed of materials that guarantee its functionality and application. The polymers used were chosen according to their characteristics. Gel, for example, is a collagen-derived biopolymer and has relevant properties such as non-toxicity, biodegradability and biocompatibility PVA; it is a synthetic, biodegradable and low-toxic polymer with excellent filmogenic properties [30]. The composition of the biofilm is an advantage of this system, as it is produced from biodegradable polymers, which allows its degradation to be rapid, minimizing damage to the environment; after use, it can be autoclaved and discarded. l-Lactic acid is one of the main volatiles secreted by the eccrine sweat glands of vertebrates and plays an important role in attracting mosquitoes. This was the first substance with an attractive effect for mosquitoes in laboratory behavior tests to be isolated and identified [31]. In double-choice olfactometer studies, *Cx. quinquefasciatus* was attracted to l-lactic acid [17, 18].

As Table 1 shows, the presence of EDTA does not significantly alter the properties of the material since the rupture force values did not change significantly; however, the Young's modulus (also known as a modulus of elasticity) presented a higher value for the films after immersion in EDTA [32]. Furthermore, the addition of EDTA to the matrix may interrupt the inter- and intramolecular hydrogen interactions between the Gel and PVA matrix, making the chains freer, thus having more elasticity [22]. These results are in agreement with the elongation of the material, which presented a slightly higher value for the biofilm after immersion in EDTA. The biofilm with EDTA preserves high elasticity, which makes it easier to handle during application. This characteristic allows the biofilm to be stretched under the blood without breaking; it also ensures that the polymeric matrix does not absorb food, allowing insects to feed [33].

Several factors can influence the feeding of Culicidae, such as tactile, visual and olfactory stimuli, temperature of the feeding surface and blood temperature, among others [27]. The commonly used way to attract females to artificial devices is to simulate the presence of the host through heat, heating the blood confined in the reservoir, mainly through water circulation systems [12, 28, 34, 35]. Devices that do not have water circulation keep the blood warm by supplying the internal compartments with heated liquid [23], a thermal bag [36], a ceramic with an electrical connection for heating [37] or electronic equipment with a metal plate [38]. Based on dual-choice olfactometer studies, *Cx. quinquefasciatus* was attracted to l-lactic acid [17, 18]. The biofilm developed in this study contains this chemical compound, which works as an attractant for mosquitoes, simulating the presence of a host, and its use in heated blood promotes an increase in the blood meal intake of *Cx. quinquefasciatus* females and shows high laboratory engorgement rates. Obtaining a number much higher than those found in the literature, Moutailler et al. [39] studied *Cx. quinquefasciatus* fed artificially using Parafilm-M^®^ membrane and obtained 1% engorged females. Other experiments carried out with the same species, using devices with heated water circulation, showed a variation in the average feeding rate between 45.0 and 48.0% [34].

Novak et al. [40], comparing different types of membranes for artificial feeding of mosquitoes, observed that *Cx. quinquefasciatus* females are more reluctant to feed through artificial membranes than directly on live animals. In our study we observed a very low feeding rate through Parafilm-M^®^, only 20%, and no eggs were laid. Dias et al. [41] evaluated the efficiency of different membranes, namely collagen, latex and Parafilm-M^®^, in feeding this culicidae and obtained percentages of fed females of 65%, 60% and 80%, respectively, but the fertility rate was only 55 to 80 eggs, with Parafilm-M^®^ being the method with the highest fertility rate. Dias et al. [34] evaluated artificial feeding blood sources for *Cx. quinquefasciatus* and observed that citrated rabbit blood presented the best result with an average of 44 eggs compared to citrated sheep blood (average of 39 eggs), defibrinated sheep blood (average of 30 eggs) and defibrinated rabbit blood (average of 36 eggs). The average direct feeding in guinea pigs was slightly higher (55 eggs). In this study, preliminary data showed that the laying rate of those fed with biofilm was 90% with an average of 158 eggs per female with anticoagulated bovine blood. This result demonstrates a satisfactory feeding rate and also that the biofilm does not interfere with the development of oocytes, allowing large egg production by females. Lima et al. [42] describe that studies with mosquitoes can use the first oviposition as a measure of fecundity.

In addition to satisfactory fecundity using the biofilm, this method also ensured high hatchability rates reaching 97%, larvae developed without major losses, and 95% reached the pupal stage. The adult emergence rate corresponded to 93%, that is, each female was capable of producing an average of 134 healthy adults. Therefore, no interference of biofilm components was observed in any developmental stage of the cycle of *Cx. quinquefasciatus*. Laboratories that produce mosquitoes on a large scale need updated protocols, with alternatives for improving colonies, such as speed in the insect feeding process, less loss of adult mosquitoes, greater egg productivity after blood meals and, consequently, greater production of mosquitoes [12]. Our artificial hematophagy process with attractive polymeric biofilm can guarantee high production rates of fertile eggs with a significant percentage reaching the adult stage. Currently, our colony of *Cx. quinquefasciatus* is in the F.9 generation, the insects are acclimatized, and the artificial hematophagy process optimizes the maintenance of the biological cycle in the laboratory.

## Conclusions

The artificial hematophagy system, featuring an attractive polymeric biofilm, is effective, practical and biodegradable, making it applicable to other species of hematophagous insects. This biofilm demonstrated a satisfactory engorgement rate and possesses suitable mechanical properties for its intended applications. Its hydrophobic nature ensured stability during the testing period.

Our study presents preliminary data demonstrating the potential of the polymeric biofilm with an attractant in the artificial feeding of this culid species in the laboratory. Further studies will be conducted with the biofilm and different systems, potentially contributing significantly to research on behavior, inter- and intraspecific interactions, environmental control and vectorial capacity, particularly regarding the maintenance of the biological cycle in the laboratory.

## Data Availability

The datasets supporting the conclusions of this article are included whithin the article.

## Abbreviations

ZIKV: Zika virus
INPI: National Institute of Industrial Property
ASTM: American Society for Testing and Materials
EDTA: Ethylenediamine tetra acetic acid
PVA: Polyvinyl acetate
